# Post-Exercise Protein Trial: Interactions between Diet and Exercise (PEPTIDE): study protocol for randomized controlled trial

**DOI:** 10.1186/1745-6215-15-459

**Published:** 2014-11-24

**Authors:** Abdullah F Alghannam, Kostas Tsintzas, Dylan Thompson, James Bilzon, James A Betts

**Affiliations:** Human Physiology Research Group, Department for Health, University of Bath, Claverton Down, Bath, BA2 7AY UK; School of Life Sciences, Queen’s Medical Centre, Nottingham, NG7 2UH UK

**Keywords:** Nutrition, Protein, Recovery, Aerobic training, Adaptation

## Abstract

**Background:**

Performing regular exercise is known to manifest a number of health benefits that mainly relate to cardiovascular and muscular adaptations to allow for greater oxygen extraction and utilization. There is increasing evidence that nutrient intake can affect the adaptive response to a single exercise bout, and that protein feeding is important to facilitate this process. Thus, the exercise-nutrient interaction may potentially lead to a greater response to training. The role of post-exercise protein ingestion in enhancing the effects of running-based endurance exercise training relative to energy-matched carbohydrate intervention remains to be established. Additionally, the influence of immediate versus overnight protein ingestion in mediating these training effects is currently unknown. The current protocol aims to establish whether post-exercise nutrient intake and timing would influence the magnitude of improvements during a prescribed endurance training program.

**Methods/Design:**

The project involves two phases with each involving two treatment arms applied in a randomized investigator-participant double-blind parallel group design. For each treatment, participants will be required to undergo six weeks of running-based endurance training. Immediately post-exercise, participants will be prescribed solutions providing 0.4 grams per kilogram of body mass (g · kg^−1^) of whey protein hydrolysate plus 0.4 g · kg^−1^ sucrose, relative to an isocaloric sucrose control (0.8 g · kg^−1^; Phase I). In Phase II, identical protein supplements will be provided (0.4 + 0.4 g · kg^−1^ · h^−1^ of whey protein hydrolysate and sucrose, respectively), with the timing of ingestion manipulated to compare immediate versus overnight recovery feedings. Anthropometric, expired gas, venous blood and muscle biopsy samples will be obtained at baseline and following the six-week training period.

**Discussion:**

By investigating the role of nutrition in enhancing the effects of endurance exercise training, we will provide novel insight regarding nutrient-exercise interactions and the potential to help and develop effective methods to maximize health or performance outcomes in response to regular exercise.

**Trial registration:**

Current Controlled Trials registration number: ISRCTN27312291 (date assigned: 4 December 2013). The first participant was randomized on 11 December 2013.

## Background

Performing regular exercise has a multitude of health benefits that stem from cardiovascular and skeletal muscle adaptations that occur in response to the exercise stimulus. Endurance exercise induces adaptations in the cardiovascular system that allow an increase in capillary number and plasma volume expansion, supporting a greater surface area for gas exchange and the movement of blood, and thus enhancing oxygen transport to the active muscles [1]. Pronounced skeletal muscle adaptation to endurance exercise include an increase in mitochondrial content and size (mitochondrial biogenesis) and increased activity of oxidative enzymes, which collectively improve oxidative capacity [2, 3]. Accordingly, the capacity to perform daily work tasks can be improved as a consequence of endurance exercise.

Adaptation to exercise is thought to occur as a consequence of the accumulated response of acute exercise bouts, while nutrient availability can influence the acute response to exercise and may modulate chronic adaptations to exercise training [4]. Therefore, nutritional strategies provided in close temporal proximity to exercise have the potential to improve training efficiency by enhancing the magnitude of adaptations to the same training stimulus [5]. Emerging acute mechanistic evidence supports the potential benefit of post-exercise protein feeding in increasing muscle protein synthesis and mitigating proteolysis associated with an endurance exercise bout [6–8]. Cycling-based endurance training studies in 2009 and 2011 have also proposed the role of protein ingestion in supporting tolerance to intensified training in concurrence with an increase in the magnitude of training adaptations when repeated bouts of exercise are performed [9–11]. Taken together, post-endurance exercise protein intake may provide means to facilitate aerobic training adaptations. Nonetheless, the time course effects of prolonged post-exercise protein co-ingestion in enhancing the adaptive response to running-based endurance exercise training in adults remains to be established.

Protein synthesis is an outcome of a complex physiological process by which external stimuli, such as contractile activity and nutrient availability, promotes phenotypic characteristics in skeletal muscle [4]. It is becoming clear that both the exercise stimulus and protein feeding act independently and synergistically in modulating muscle protein synthesis, and consequently these subtle changes in muscle quantity or quality can mediate worthwhile training adaptations if sustained for weeks or months [4, 12]. Furthermore, protein ingestion during and following an acute endurance exercise bout has been shown to increase muscle protein synthesis and reduce muscle protein breakdown, and thus results in an increased whole body net protein balance [6, 13]. In spite of the fact that endurance exercise does not typically result in muscle mass accrual, the changes in muscle protein synthesis following endurance exercise are relevant to drive tissue repair and remodeling, in concurrence with the synthesis of non-contractile proteins, such as the mitochondria [14]. Indeed, acute and chronic endurance exercise has been demonstrated to stimulate the mitochondrial protein synthetic response [15, 16]. While the influence of protein ingestion on mitochondrial biogenesis machinery is not clear at present [8, 11, 17], its synthesis appears to be stimulated by the availability of extracellular amino acids in a dose-response manner [18]. This may infer that exogenous protein availability can enhance the capacity for muscular adaptations, although the role of post-exercise protein ingestion in enhancing the effects of running-based endurance exercise training remains largely unknown [11, 19].

Cardiovascular adaptations are a hallmark of endurance exercise training. An enhancement in oxidative capacity and maximal oxygen uptake () in response to aerobic training with post-exercise protein ingestion has been observed by some [10, 11], but not all [20] studies. In concordance, the role of protein feeding in enhancing the adaptive response to endurance training may not reside in the intramuscular milieu. Rather, noticeable cardiovascular improvements in  via an increase in plasma volume and plasma albumin content have been shown with protein ingestion [10, 11]. However, the addition of other nutrients such as caffeine, flavonoids, multivitamins and ribose [11, 20], in addition to the absence of macronutrient-specific comparisons by not including a carbohydrate-only supplement [10], makes it difficult to conclude whether these improvements in training adaptation are a result of protein intake *per se*. Interestingly, the magnitude of improvement in  during endurance training was greater when whey protein was ingested relative to a placebo [10], or when milk-based protein available in the form of chocolate milk was co-ingested, when compared to an energy-matched carbohydrate supplement and a placebo [11]. Collectively, these findings may infer nutrient-specific effects of post-exercise supplementation on endurance training adaptations.

In summary, recent scientific evidence from acute laboratory investigations and relatively extended cycling-based training studies supports the notion that nutrient intake can increase protein accretion and, ultimately, this may influence the magnitude of the training effect. This nutrient-exercise interaction may modulate the adaptive response to training and protein feeding appears to be an important factor in mediating this process. However, examining the long-term outcomes of these acute mechanistic models, particularly during free living endurance-type exercise, require further investigation. This project aims to address two issues (Phase I and Phase II). Phase I will address whether protein co-ingestion with carbohydrate immediately following exercise can increase the magnitude of cardiovascular and intramuscular training adaptations to endurance training when compared with ingestion of carbohydrate alone. Phase II while address to what extent the timing of protein co-ingestion (overnight versus immediate feeding) impacts upon those training adaptations.

It is hypothesized that the inclusion of protein in a post-exercise nutritional supplement will increase the magnitude of improvement in cardiovascular, but not intramuscular, adaptations following a long-term endurance training intervention in Phase I. Furthermore, it is hypothesized that overnight protein feeding will enhance the intramuscular adaptive response (markers of mitochondrial biogenesis) to endurance training relative to immediate feeding in Phase II.

## Methods/Design

### Approach to research questions

The primary outcomes of this trial are the assessment of cardiovascular (changes in , plasma albumin content and plasma volume) and intramuscular (selected genes and proteins involved in cellular adaptive processes) training-induced adaptations, and whether protein co-ingestion (immediate or overnight) improves the magnitude of these adaptations. In accordance, two phases of testing will be conducted.

Phase I will be addressed by evaluating a carbohydrate-protein mixture against an energy-matched carbohydrate control to examine the influence of post-exercise protein intake in enhancing endurance training adaptations. Participants in Phase I will be randomly allocated to a group receiving an isocaloric carbohydrate supplement or a group receiving a carbohydrate-protein supplement, in a randomized investigator-participant double-blind manner, whereby the chief investigator (AFA) and participants will be blinded from the trial allocation. The acute post-exercise phase is important when considering the metabolic priority for recovery to be initiated [3, 21] and the potentiated sensitivity to nutritional interventions during this period [22]. It has been established that glycogen resynthesis and amino acid uptake rates are augmented following an exercise bout, and that the ingestion of carbohydrate and protein stimulate this process [6, 23–25]. This has subsequently prompted large numbers of studies to investigate the role of combined carbohydrate-protein ingestion in restoring glycogen stores, potential attenuation of decrements of skeletal muscle functional integrity and subsequent exercise performance [12, 26]. It is now generally accepted that incorporating protein into a post-exercise feeding regimen is a viable strategy to enhance recovery, mainly through observations in acute based studies [7, 8, 21, 27–33]. Yet, the effects of protein ingestion are more likely to be realized beyond the acute recovery phase, given that upregulation of endurance exercise-specific gene expression peaks between 10 and 24 hours following an exercise bout, and may surpass 96 hours for muscular recovery and adaptive remodeling to occur, suggesting that repeated bouts of exercise are required to detect worthwhile training adaptations [3, 34].

Given that the target population will be physically untrained and subjected to running-based aerobic exercise training, and that reversal of these training-induced adaptations are relatively slow in such individuals [35], the study will adopt a randomized parallel group design in each phase with a relatively larger cohort of participants to account for inter-individual variability. The duration of the training period will be six weeks. This duration was deemed adequate based on previous literature, which demonstrated that approximately four weeks (total number of sessions was between 20 and 22 during the entire protocol) elicits changes in  of 6 to 14%, oxidative enzyme activities of 35 to 50% and mitochondrial protein content of 50 to 100% in untrained and moderately trained individuals [11, 36, 37].

Phase II of testing will examine the influence of protein timing upon mediating the adaptive response to endurance exercise. Participants will be randomly assigned to a group receiving the same carbohydrate-protein mixture in Phase I, but with one group ingesting the supplement immediately post-exercise, while the second group supplementation will take place during overnight recovery in a double-blinded manner. The interaction between exercise and protein ingestion on mitochondrial protein synthesis may lie outside the acute recovery phase (over eight hours post-exercise) and under circumstances of rested mitochondrial turnover rates [8, 19]. Moreover, the provision of dietary protein prior to sleep was shown to be an effective nutritional strategy to further augment muscle protein synthesis during overnight recovery [38]. Coupled with the fact that exogenous amino acids stimulate mitochondrial synthetic response in the rested state [18], protein intake during overnight recovery may increase the adaptive response to endurance training relative to immediate feeding. Therefore, comparisons between immediate versus overnight protein ingestion (Phase II) are warranted to provide an insight into the molecular and transcriptomic responses that could extend the knowledge regarding the relationship of nutrient-exercise stimuli to mitochondria biogenesis.

It has also been established that rapid hyperaminoacidemia (particularly leucine) is required to maximize muscle protein synthetic response; however, this response is returned to baseline approximately three hours after a single bolus of ingested protein within the recommended quantities (approximately 0.3 to 0.4 grams per kilogram of body mass; g · kg BM^−1^) for maximal muscle protein synthesis [39–41]. Therefore, it may be postulated that a second bolus would be advantageous in inducing hyperaminoacidemia and would supply sufficient amounts of leucine into circulation to prompt the ‘leucine trigger’ [40, 42, 43], consequently maximizing muscle protein synthesis during post-exercise overnight recovery.

### Participants

A total of 32 research participants will be recruited for each phase from the local community via public advertisement (including online posts and distribution of flyers) and personal communication. Upon contacting the chief investigator (AFA), participants will be provided with a participant information sheet detailing the study aims and requirements. Participants who express their interest will be scheduled for an initial meeting to assess their eligibility and provide further details regarding the study requirements. An informed consent form will be obtained from individuals who agree to participate following the briefing, indicating their full understanding of the study and their protected rights for confidentiality and withdrawal from the study. Thereafter, a medical health questionnaire will be undertaken by each participant to ensure the absence of any physical, hematologic, metabolic or any other health conditions. If any of the factors above are present, the volunteer will be deemed unfit to participate in the study and will consequently be excluded from taking part. Further health screening and a patient-specific direction for the use of anesthetic will be completed and sent to a medical practitioner, who will respond to confirm the absence of any contraindications prior to any local anesthesia administration.

The protocol described herein was reviewed and approved by the National Health Service (NHS) South West 3 Research Ethics Committee (approval number: 13/SW/0239). The project was subsequently registered as a controlled trial (Current Controlled Trials registration number: ISRCTN27312291). The individuals who participate in Phase I of testing will not be eligible to take part in Phase II.

### Inclusion criteria

The inclusion criteria for this study are as follows:

1. Both male and female volunteers.
2. Aged between 18 and 48 years.
3. Individuals free from cardiovascular, metabolic or joint disease as determined by a standard health questionnaire.
4. No engagement in structured physical activity for more than two hours per week during the last two years.
5. Nonsmoker.

### Exclusion criteria

The exclusion criteria for this study are as follows:

1. Known or suspected food intolerances, allergies or hypersensitivity.
2. Any bleeding disorder or taking medication which impacts blood coagulation.
3. Known tendency towards keloid scarring.
4. Known sensitivity or allergy to any local anesthetic medicines.
5. Any reported use of substances which may pose undue personal risk to participants or introduce bias into the experiment.
6. Any other condition or behavior deemed either to pose undue personal risk to participants or introduce bias into the experiment.

The sex of participants will be included in a stratified randomization scheme using a computer-based random number generator produced by an academic supervisor (JAB), who is not responsible for trial enrollment, nutritional preparation or provision. The only interaction by JAB with participants will be for obtaining muscle biopsies at baseline and follow-up, and thus he will be unaware of the assigned coding numbers to participants, rendering him unable to determine trial allocation to any participant. Details of the overall randomization scheme will only to be published once follow-up measurements are completed in order to complicate deciphering of the allocation sequence by those involved in trial enrollment [44].

### Experimental design

The proposed project involves two distinct phases of testing, each contrasting two nutritional interventions in a randomized investigator-participant double-blind parallel group design. The independent variable will be the precise nutritional intervention ingested following exercise in Phase I, while timing of ingestion will be the independent variable in Phase II. Specifically, those participants recruited for Phase I (n =32, approximately) of testing will receive a supplement containing carbohydrate (sucrose) plus protein (whey protein hydrolysate) at an ingestion rate of 0.4 and 0.4 g · kg BM^−1^ respectively. The second group will be a control group, whereby an ingestion of an isocaloric carbohydrate supplements will be assigned (0.8 g · kg BM^−1^). The two nutritional interventions provided in Phase II (n =32, approximately) will be identical (0.4 g · kg BM^−1^ carbohydrate +0.4 g · kg BM^−1^ whey protein hydrolysate), while the timing of intake will be manipulated. A group will ingest the carbohydrate-protein supplement immediately post-exercise and one hour later, while the overnight recovery group will receive the supplements one hour prior to sleep, and at 02:00, as an overnight nutritional intervention. The nutritional information for the supplements is provided in Table 1.

**Table 1:** **Nutritional information of the supplements provided in Phase I and Phase II of the trial**

	High sucrose	Sucrose + whey protein hydrolysate
**Sucrose (g/l)**	80	40
**Lactose (g/l)**	-	≤2.3*
**Protein (g/l)**	-	40
**Fat (g/l)**	-	≤2.4*
**Sodium (g/l)**	-	0.72
**Potassium (g/l)**	-	0.10
**Calcium (g/l)**	-	0.15
**Magnesium (g/l)**	-	0.01
**Phosphorous (g/l)**	-	0.19
**Chloride (g/l)**	-	0.29
**Energy (kcal/)**	320	320

*Assay unable to detect values below this number. The caloric content for fat and lactose was therefore assumed to be negligible.

Supplements will be provided in a sachet form along with shaker bottles with measurement scales. Solution preparation will be instructed to participants to achieve a volume of ingestion of 10 ml · kg BM^−1^. The entire protocol will be eight weeks duration and will consist of: (i) a baseline testing week with two exercise sessions, (ii) the first, second and third weeks of training at 70% , (iii) the fourth, fifth and sixth weeks of training at 75%  and (iv) a final week for follow-up measurements. A total of 26 endurance-based training sessions will be prescribed for each participant.

### Baseline testing

Prior to the start of the training period, participants are required to attend the laboratory on two occasions. The first visit will include anthropometric assessment of height, weight and body mass. Participants will also undergo an assessment of their running economy ( · km^−1^) and  (details provided below). The second preliminary visit will then be arranged within a week following the first laboratory visit. A 48-hour standardization of lifestyle will be employed (discussed below). This visit will involve obtaining fat mass percentage using bioelectrical impedance analysis (BIA), followed by a resting expired gas sample to measure resting metabolic rate (RMR). After laying on a semi-supine bed for five minutes, two five-minute baseline resting expired gas samples will be taken for the estimation of RMR, along with resting heart rate via short-range telemetry (Polar FT2, Kempele, Finland). A venous blood sample (10 ml) will also be obtained from an antecubital vein to measure different plasma metabolites, in addition to hematological parameters for the estimation of plasma volume change. Subsequently, an 80 to 100 mg muscle biopsy sample will be obtained from the vastus lateralis under a local anesthetic (1% lidocaine; Hameln Pharmaceuticals Ltd., Brockworth, United Kingdom). In relation to eumenorrheic female participants, all measurements will be conducted three to 10 days after the onset of menses (follicular phase) to ensure low levels of circulating female hormones and therefore minimize any measurement errors associated with menstrual cycle [45]. Two exercise sessions will then be employed during the baseline testing week that require participants to run on a motorized treadmill for 30 minutes per day. These were aimed to allow participants to gauge the relative intensity required during their prescribed training intervention, while also familiarizing them with treadmill running, before the commencement of the six weeks training intervention.

### Training intervention

Exercise sessions performed in the baseline testing week will last for 30 minutes and thereafter the duration of sessions will progressively increase to 40, 50 and 60 minutes in week one, weeks two to three, and weeks four to six, respectively. Prior to any exercise session, a five-minute warm-up at 60%  will be superimposed, followed by treadmill running at a speed corresponding to 70%  for the durations indicated above. The target %  will be attained by providing participants with their relative speeds at any given intensity during the training intervention, which will be determined by linear regression (Excel 2010, Microsoft, Redmond, Washington, United States) during the first baseline visit. During the midpoint (training week three), the speed will be increased to elicit 75% . Only water consumption will be permitted during exercise sessions, which will be consumed *ad libitum*. Upon cessation of each exercise session in Phase I, a post-exercise nutritional supplement will be ingested immediately, while a second bolus will then be consumed one hour after the end of exercise. In relation to Phase II of testing, one group will ingest a post-exercise nutritional supplement in a similar manner to Phase I, while the other group will ingest an identical supplement one hour prior to sleep and at 02:00. During the nutritional provision periods, participants will be instructed to consume only water to avoid any confounding results relating the outcome variables of the study. Once every fortnight, participants will report to the laboratory to provide the nutritional supplements for the subsequent two-week training block, with a total of three scheduled meetings throughout the training intervention. This visit is also to confirm the adherence of participants to the prescribed training and supplementation procedures.

Although several risk factors will be screened before participants take part in exercise, the prescribed physical exercise may increase the risk of certain adverse events (such as myocardial infarction, musculoskeletal injuries and so on). Nevertheless, the prescribed endurance training will be conducted in a gymnasium under supervision of trainers who are familiar with first aid procedures, and the occurrence of any adverse events related to the study will be reported.

### Follow-up testing

**Figure 1:**
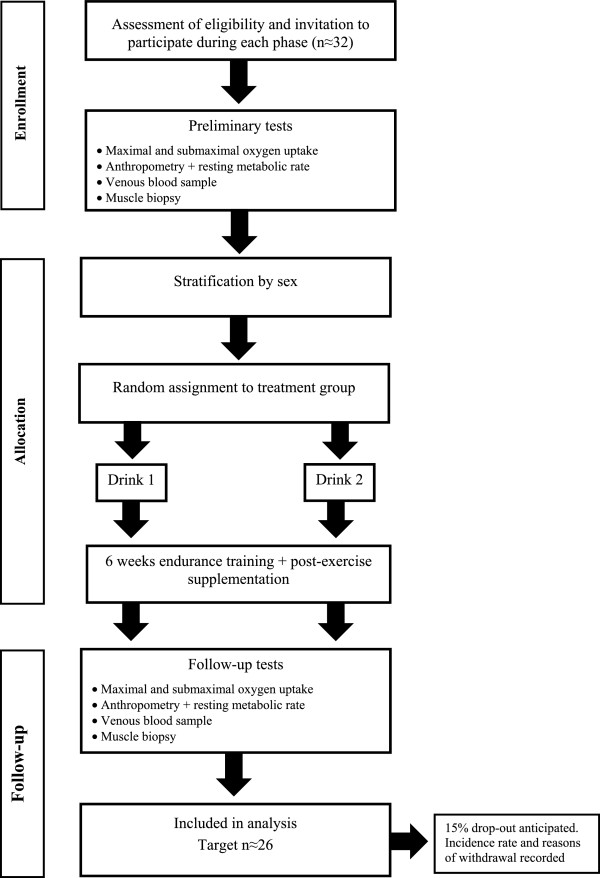
**Flow diagram of study protocol.**

The follow-up procedures after the training intervention will be identical to those in the baseline testing phase of the experiment (Figure 1). Importantly, the interval between the final session of prescribed training and follow-up measurements will be kept as close as possible to avoid any de-training effects [35]. Namely, measurements of running economy and  will be taken at least one, and at most two days, following the final exercise training session. This will be followed by 48 hours of standardization of lifestyle (replicating procedures of dietary records and activity obtained two days before baseline measurements) before obtaining any measurement pertaining to the second follow-up laboratory visit, a period of sufficient length to separate the chronic effects of training from exercise-induced responses [46].

### Anthropometry

The determination of post-void nude body mass for the participants will be recorded on their first visit to the laboratory to allow for an accurate nutrient provision for each participant during the training intervention that are calculated relative to each participant’s body mass. Weighing of each participant’s body mass will be undertaken using a balance scale (Weylux 424, Fereday & Sons Ltd., United Kingdom), while the measurement stature will be recorded using a stadiometer (Holtain Ltd., Crosswell, UK) following a protocol described elsewhere [47]. From the collective measurements obtained above, body mass index will then be determined (kg · m^−2^).

Body fat percentage (%BF) estimates will be taken during the second laboratory visit at baseline and follow-up testing, each following 48 hours of standardized lifestyle control. These measurements will be based on BIA analysis method (BC-543, Tanita, Tokyo, Japan). This method was shown to overestimate %BF by 3 to 4% in healthy lean males and females (<15% and <25%BF, respectively) and underestimate %BF by 3 to 4% in overweight/obese males and females (>25% and >33%, respectively), in a healthy population, relative to the dual-energy X-ray absorptiometry reference method [48]. While changes in %BF are not anticipated in the current study protocol based on findings from similar investigations [11, 20], the bias in BIA measurement will be taken into consideration when interpreting or discussing any data related to %BF in the current protocol [48].

### Submaximal and maximal oxygen uptake measurements

Running economy (· km^−1^) and () measurements will be assessed for participants during the first laboratory visit during baseline and follow-up testing [49]. These tests initially require participants to run on a motorized treadmill (Ergo ELG70, Woodway, Weil am Rhein, Germany). The protocol involves a standardized five-minute warm-up that consists of jogging at a speed of 7.5 km · h^−1^, followed by a running economy test. This requires participants to run at various submaximal speeds with increments of 1 km · h^−1^ to enable a minimum of four different running speeds (8 to 11 km · h^−1^). Each running speed will consist of three-minute stages, during which expired gas, heart rate (HR) and ratings of perceived exertion (RPE) will be recorded at the final minute of each stage. Expired gas samples, HR and RPE will be obtained via the Douglas bag method, short range telemetry (Polar FT2, Kempele, Finland) and Borg’s scale (range of the scale: 6 to 20) [50], respectively. Approximately 20 minutes following the submaximal test, a run time to volitional exhaustion will be initiated to determine relative  values for participants, which is described in further detail elsewhere [47]. The criteria of the British Association of Sport and Exercise Sciences for establishing  in adult subjects will be used to ensure the attainment of  [51]. These will include the achievement of a maximal HR within 10 beats · min^−1^ from the predicted HR maximum for participants (220 beats · min^−1^ − age), respiratory exchange ratio (RER) values of 1.1 or greater and subjective indication of volitional exhaustion on the RPE scale. The data acquired from these tests will subsequently be used to calculate the treadmill speeds required by the trial procedures (speeds that elicit 60, 70 and 75% ) by linear regression (Excel 2010, Microsoft, Redmond, Washington, United States).

### Physiological measurements

#### Hydration

During baseline and follow-up measurement days, urine samples will be collected to determine hydration via freezing point depression method, using a cryoscopic osmometer (Advanced Instruments, Inc, Norwood, Massachusetts, United States). The threshold for adequate hydration will be assumed for osmolality values ≤900 mOsm · kg^−1^ [52].

#### Expired gas sampling

The Douglas bag method (Hans Rudolph, Shawnee, Kansas, United States) will be used for expired gas analysis. A respiratory valve with a mouth piece will be attached to a 200 l Douglas bag for expired gas sampling and provided with a nose clip to participants approximately 30 seconds before sampling to remove any residual atmospheric air from the valves. The collected gas samples will then be analyzed for relative expired fractions of oxygen and carbon dioxide using a paramagnetic and infrared analyzers, respectively (Servomex, Crowborough, United Kingdom). The total volume of expired gas within the Douglas bag will subsequently be measured by a dry gas meter (Harvard Apparatus, Kent, United Kingdom), with the temperature of expired gases being collected at the time of evacuation by a thermistor probe (Grant Instruments Ltd., Cambridgeshire, United Kingdom). Calibration of equipment will be conducted prior to any measurements using gas cylinders containing specific gases with a known relative composition (N_2_ = 0%; O_2_ = 16.9%; CO_2_ = 4.93%), as validated by the manufacturer (CryoService, Worcester, United Kingdom). Each analyzer will then be validated against atmospheric air bottled within a Douglas bag. Inspired air will be measured proximally to the participants before obtaining any corresponding expired gas sample, in order to minimize bias associated with assumptions that inspired gas fractions are stable and reflective of atmospheric constants [53].

Indirect calorimetry based on calculations of oxygen consumption and carbon dioxide production from each bag will then be used to obtain measurements of RMR. The processes followed by individuals being measured and those to be applied by the researchers will follow recommendations by The American Dietetic Association related to best practice for measuring RMR [54]. The processes followed include participants arriving from an eight to 10-hour overnight fast following two days of standardized physical activity and diet in a quiet, private space with temperature controlled between 20 and 25°C. Additionally, participants will be rested in the same reclined position for five to seven minutes before steady-state conditions and measurement interval (two five-minute periods) occur.

#### Blood sampling

Venous blood samples will be obtained by venipuncture using an antecubital vein. Prior to any attainment of a blood sample, participants will be rested in a semi-supine position for 15 minutes. A 10 ml blood sample will be collected before being dispensed into two 5 ml EDTA-treated tubes (Sarstedt, Leicester, United Kingdom). The first of these tubes will be analyzed for hemoglobin concentrations by using an automated hematology analyzer (Sysmex SF-3000; Sysmex Ltd., Wymbush, United Kingdom). Thereafter, three 50 μl aliquots of blood will be removed using micro-hematocrit tubes, and subsequently centrifuged (Hawksley, Lancing, United Kingdom) to obtain hematocrit concentrations. Hemoglobin and hematocrit concentrations will then be used to determine changes in plasma volume [55]. The remaining EDTA-treated blood will then be spun for centrifugation under 2000 xg for 10 minutes at 4°C (Heraeus Primo R; Thermo Fisher Scientific, Loughborough, United Kingdom) for plasma extraction then stored at −80°C for later analysis of plasma albumin concentration utilizing an automated spectrophotometric analyzer (RX Daytona, Randox, Crumlin, Ireland). Prior to each sample analysis, a calibration will be conducted and quality control will be checked against manufacturer’s available standards. Plasma albumin content will consequently be determined from plasma albumin concentration and the change in plasma volume, as previously described [56].

#### Muscle biopsy sampling

An 80 to 100 mg muscle biopsy sample will be obtained at baseline and follow-up via needle biopsy techniques [57]. A 5 mm skin incision will be made from the lateral portion (vastus lateralis) of the thigh from the same leg at baseline and follow-up and separated by 2 to 3 cm, with the use of dominant and non-dominant legs being counterbalanced between participants. The detailed procedures for obtaining the muscle biopsy samples are reported elsewhere [47].

Once removed from the thigh, each muscle sample will be immediately immersed in liquid nitrogen and kept stored pending subsequent analysis. The obtained muscle samples will be freeze-dried and dissected free of any visible adipose, blood and connective tissue, and separated into two specimens with a scalpel. One part of the frozen muscle tissue will be used to evaluate the impact of endurance training on selected genes and proteins involved in cellular adaptive processes (such as *GLUT-4, HKII, FABPpm, PDK4, PPARδ, PGC-1α, SIRT1, AMPK, P38MAPK, P53, ERK1* and *2*) using quantitative real-time PCR and Western blotting, as previously described [58]. Another part of the frozen muscle tissue will be utilized to determine the enzyme activity of citrate synthase [59].

#### Standardization of lifestyle

Throughout the duration of the study, participants will be asked to maintain constant dietary energy intake and activity levels (no important changes in caloric intake, dietary habits or physical activity levels outside the scope of the prescribed supplements and training sessions). The prescribed exercise training is anticipated to only result in a small degree of dietary compensation by increasing the energy intake to offset the increase in energy expenditure, with no compensatory reduction in non-prescribed physical activity, particularly in view of the free-living conditions introduced by the study [60]. Over the 48 hours prior to baseline testing, a weighed dietary record and an exercise activity log will have been completed by participants. These dietary records will be analyzed using nutritional analysis software (Nutritics LTD, Dublin, Ireland) and reported as the average total daily energy intake and percentage contribution of carbohydrate, protein and fat. The exercise activity logs over the 48-hour period will be assessed to examine the mode of exercise (for example cycling, running or resistance exercise) and total minutes of activity during a given exercise session. Participants will then be required to replicate these procedures in the 48 hours preceding the follow-up testing. Alcohol abstinence will be employed throughout this period, while caffeine consumption will be permitted until 17:00 on the day preceding any testing to avoid any unnecessary side effects as a result of adverse withdrawal from caffeine consumption [61]. Moreover, three, three-day dietary records will be assessed and subsequently analyzed from participants at different intervals during the six-week training intervention period. This aims to provide a reflection of dietary intake habits between groups during the training period.

During prescribed training days, participants will be required not to perform any other structured exercise session and will be instructed to ingest a meal (breakfast or lunch, depending on the time of day) approximately two hours prior to any session, and thereafter abstain from any caloric intake prior to the commencement of the training session. Participants will also be instructed not to consume any calories outside the scope of the prescribed supplements during the designated supplement provision periods (either two hours immediately post-exercise or one hour prior to sleep and throughout the overnight period, depending on the phase of testing). A checklist will be included in Phase I and Phase II of testing to confirm supplement intake, time of ingestion and avoidance of any caloric intake outside the scope of the prescribed supplements. This is aimed to ensure adherence to the nutritional intervention intake and timing throughout the training period.

In an attempt to accurately monitor training and ultimately standardize the training load between treatments *in situ*, an exercise diary will be provided to each participant. Participants will complete the relevant information relating to the exercise session (time of day the training session was started, total duration of session) on any of the training days, which will then be verified by an electronic monitoring system that requires participants to use a key card to enter and leave the gymnasium. Once the study is completed, the exercise logs will be collected and cross-examined with the electronic monitoring system to verify adherence for each exercise session attendance, for each date and duration.

#### Environmental measurements

Ambient temperature, humidity and barometric pressure will be monitored and recorded throughout the trials using a portable weather station (WS 6730; Technoline, Germany). The latter will be used to record atmospheric pressure to allow for corrections to standard volumes during expired gas analysis.

### Sample size estimation

*A priori* sample size estimation (G*power version 3.1.7, University Düsseldorf, Düsseldorf, Germany) from data in the literature similar to the proposed work [11], revealed that a total of 24 participants are required to achieve 90% power to detect a worthwhile increase in an endurance performance () of 5.3 ml · kg^−1^ and standard deviation of 3.3 ml · kg^−1^, using a two-tailed t-test for independent means with an alpha level of 0.05. Rolling recruitment of 32 participants in each phase of testing will therefore be conducted when considering an anticipated 15% dropout rate [11, 36].

### Data analysis

The majority of data include measurements of cardiovascular and intramuscular markers of endurance training adaptations at baseline and follow-up, and thus treatments × time interactions will be explored using a general linear model with baseline included as a covariate, to account for any baseline imbalances because of regression to the mean [62]. The majority of variables in this experiment therefore involve a single comparison between two level scores, and so will not be adjusted for multiple comparisons across the different variables. Any data that require a single comparison of two means will be tested for normality using the Shapiro-Wilk test before using an independent t-test or non-parametric equivalent to examine differences between treatment arms (for example, magnitude of change in absolute and relative  between treatments). Relationships between outcome variables will be examined using a Pearson’s product-moment correlation and any meaningful associations (r ≥0.7) between baseline, and the magnitude of response to exercise training will be further explored by post-stratification of the treatment group according to baseline status. Significance will be set at ≤0.05 and all results being reported as the mean and standard error of the mean (SEM) unless stated otherwise.

Participants will be advised at the initial trial enrollment meeting to carefully consider the required investment of time and effort involved in the current project. This will be undertaken to minimize any negative impact associated with loss to follow-up, and consequently likely withdrawal can be made prior to randomization [63]. However, any unforeseen circumstances or withdrawal resulting in loss to follow-up will be documented and reported in the final trial report. In accordance, the handling of missing data subsequent to withdrawal will be primarily approached in a complete-case analysis adjustment method, while an imputation method will be considered where appropriate [64, 65].

## Discussion

The positive effects of supplementing protein in a post-exercise feeding regimen are clearly established in acute-based investigations in promoting recovery and, albeit acutely, the adaptive response through an increase in muscle protein accretion. At present, the long-term outcomes for such nutritional strategies remain poorly understood despite the potential influence of nutrient intake in maximizing the adaptive response to exercise training. The current project aims to investigate the role of post-exercise nutrient intake composition and timing in mediating the magnitude of adaptation to endurance exercise training. This will provide novel insight regarding nutrient-exercise interactions and its potential to help and develop effective and efficient means for improving human health or performance in response to regular exercise participation.

## Trial status

The first participant was randomized on 11 December 2013. Rolling recruitment and data collection for Phase I has commenced. Phase II recruitment is forthcoming.

## Abbreviations

%BF: Percentage body fat
BIA: Bioelectrical impedance analysis
EDTA: Ethylenediaminetetraacetic acid
HR: Heart rate
PCR: Polymerase chain reaction
RER: Respiratory exchange ratio
RMR: Resting metabolic rate
RPE: Ratings of perceived exertion
*GLUT-4*: glucose transporter type 4
*HK*: mitochondrial hexokinase II
*FABPpm*: plasma membrane fatty-acid binding protein
*PDK4*: pyruvate dehydrogenase kinase 4
*PPARδ*: peroxisome proliferator-activated receptor delta
*PGC-1α*: peroxisome proliferator-activated receptor gamma coactivator 1-alpha
*SIRT1*: sirtuin 1
*AMPK*: 5′ adenosine monophosphate-activated protein kinase
*P38MAPK*: P38 mitogen-activated protein kinase
*P53*: tumor suppressor p53
*ERK1* and *2*: extracellular signal-regulated kinase 1 and 2
